# Epithelial ovarian cancer risk: A review of the current genetic landscape

**DOI:** 10.1111/cge.13566

**Published:** 2019-05-29

**Authors:** Nicola Flaum, Emma J. Crosbie, Richard J. Edmondson, Miriam J. Smith, Dafydd G. Evans

**Affiliations:** ^1^ Division of Evolution and Genomic Sciences, School of Biological Sciences, Faculty of Biology, Medicine and Health, University of Manchester Manchester Academic Health Science Centre Manchester UK; ^2^ Manchester Centre for Genomic Medicine, St. Mary's Hospital, Manchester University NHS Foundation Trust Manchester Academic Health Science Centre Manchester UK; ^3^ Division of Cancer Sciences, School of Medical Sciences, Faculty of Biology, Medicine and Health, University of Manchester Manchester Academic Health Science Centre Manchester UK; ^4^ Department of Gynaecology, St Mary's Hospital, Manchester University NHS Foundation Trust Manchester UK; ^5^ Prevention Breast Cancer Centre and Nightingale Breast Screening Centre University Hospital of South Manchester Manchester UK; ^6^ Department of Cancer Genetics, The Christie NHS Foundation Trust Manchester UK; ^7^ Manchester Breast Centre, Manchester Cancer Research Centre University of Manchester Manchester UK

**Keywords:** BRCA, genetic risk, homologous recombination, mismatch repair, ovarian cancer, polygenic risk score, SNPs

## Abstract

Ovarian cancer is the fourth most common cause of cancer‐related death in women in the developed world, and one of the most heritable cancers. One of the most significant risk factors for epithelial ovarian cancer (EOC) is a family history of breast and/or ovarian cancer. Combined risk factors can be used in models to stratify risk of EOC, and aid in decisions regarding risk‐reduction strategies. Germline pathogenic variants in EOC susceptibility genes including those involved in homologous recombination and mismatch repair pathways are present in approximately 22% to 25% of EOC. These genes are associated with an estimated lifetime risk of EOC of 13% to 60% for *BRCA1* variants and 10% to 25% for *BRCA2* variants, with lower risks associated with remaining genes. Genome‐wide association studies have identified single nucleotide polymorphisms (SNPs) thought to explain an additional 6.4% of the familial risk of ovarian cancer, with 34 susceptibility loci identified to date. However, an unknown proportion of the genetic component of EOC risk remains unexplained. This review comprises an overview of individual genes and SNPs suspected to contribute to risk of EOC, and discusses use of a polygenic risk score to predict individual cancer risk more accurately.

## Graphical abstract

- Epithelial ovarian cancer (EOC) is one of the most heritable cancers.
- Individual gene pathogenic variants are present in 22% to 25% of cases and 6.4% of additional risk is explained by single nucleotide polymorphisms.
- A proportion of the genetic risk of EOC remains unexplained.
- Issues remain regarding use of polygenic risk scores and how best to advise women concerned about their risk of EOC.

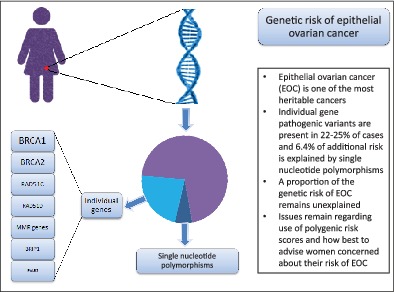

## 1 INTRODUCTION

With knowledge of risk of ovarian cancer rapidly increasing, physicians are better equipped to advise women and their families than ever before regarding their individual risk. Due to public advertisements of genetic home testing, the “Angelina Jolie effect,”^1^ general media coverage of cancer genetics and widening access to the internet and social media, the general public are becoming increasingly aware of the use of genetic testing in assessing cancer risk. However, risk assessment of ovarian cancer at the individual level is still relatively imprecise, and predominately based on environmental, familial and hormonal factors. Much is still also not known about the influence of individual genes on risk of ovarian cancer especially the contribution not explained by the *BRCA1* and *BRCA2* genes and the role of single nucleotide polymorphisms. Further research is needed to identify additional variants involved and to improve the accuracy of multifactorial risk assessment in an individual's risk of ovarian cancer to enable physicians to advise patients optimally regarding risk reduction strategies.

### 1.1 Ovarian cancer

Ovarian cancer is the fifth most common cause of cancer in women in the developed world and fourth most common cause of cancer‐related death.^2^, ^3^ It carries an estimated lifetime risk of one in 54 to 75, and one in 100 of ovarian cancer‐related mortality.^3^, ^4^ The age‐standardized incidence is approximately 9.4 per 100 000 in developed regions and 5 per 100 000 in less developed areas.^5^ Frequently diagnosed at an advanced stage, symptoms can be vague and sometimes misattributed to irritable bowel syndrome.^6^ The median age at diagnosis is 63 years.^7^ Prognosis of invasive epithelial ovarian cancer is influenced by age, International Federation of Gynaecological Oncologists (FIGO) stage, performance status, volume of residual disease after initial debulking surgery and *BRCA* status.^6^, ^8^ Median progression‐free survival (PFS) for patients with advanced ovarian cancer is approximately 18 months, and overall survival (OS) for all ovarian cancer 40% to 50% at 10 years.^6^

Epithelial ovarian cancer (EOC) comprises 60% of ovarian tumours, and is further classified into benign, borderline and malignant. High grade serous ovarian cancer (HGSOC) comprises 70% to 80% of malignant EOC, and usually presents at a late stage with disseminated disease.^9^ Originally thought to originate from the ovarian surface, these are now thought to originate predominantly from fallopian tube epithelium.^9^ Pathogenic somatic variants have been found in *TP53* in almost 100% of HGSOC tumours, and also in *FAT3, CSMD3, NF1, RAD51C, RAD51D, BRIP1, RB1, GABRA6, CDK12* and well‐known tumour suppressor genes *BRCA1* and *BRCA2*. Notch and FOXM1 signalling pathways are also implicated.^10^, ^11^, ^12^, ^13^ The genomic instability present in HGSOC promotes the development of further variants, increases genetic diversity and development of genetically distinct subclones within a tumour.^14^ Genomic instability can be associated with treatment resistance and poor prognosis if subclones develop genomic characteristics that benefit tumour survival. However, conversely, higher levels of genomic instability can enable the acquisition of pathogenic variants with a selective disadvantage, by limiting tumour growth or increasing response to chemotherapy.^14^ In HGSOC, higher levels of genomic instability are associated with higher platinum‐based chemotherapy and poly ADP ribose polymerase (PARP) inhibitor response rates, and improved survival outcomes.^14^

Low grade serous ovarian cancer makes up 10% of serous ovarian cancers. It behaves in a more indolent fashion than HGSOC, and has low response rates to chemotherapy and hormonal agents.^6^ They are commonly diagnosed at an advanced stage and OS is poor.^9^, ^15^ Women with low grade serous ovarian cancer rarely have a family history of breast and/or ovarian cancer.^16^ In contrast to HGSOC, pathogenic somatic variants have been found in *KRAS, NRAS, BRAF, ERBB2* and *PI3KCA* oncogenes.^6^ The mitogen‐activated protein kinase (MAPK) pathway is frequently activated, accomplished by variants in *KRAS* and *BRAF*.^16^

Endometrioid ovarian cancer accounts for 10% of EOC.^17^ Almost half present with stage I disease and the overall prognosis is favourable, although poor in advanced stage disease.^18^ Genomic analysis has identified pathogenic somatic variants in *ARID1A, PIK3CA, PTEN, PP2R1A* and microsatellite instability resulting from mismatch repair (MMR) deficiency.^6^ *CTNNB1* variants are very common.^9^

Clear cell ovarian cancer comprises 5% to 10% of post‐menopausal EOC^17^; women present young and there is a higher incidence in those of Asian origin and an association with hypercalcaemia.^19^ Women diagnosed at early stage have an excellent prognosis, but response rates and survival in advanced disease are poor.^17^, ^20^ The most common genetic pathogenic variants are in *ARID1A, PIK3CA, PTEN, CTNNB1* and *PP2R1A* genes,^6^ with *ARID1A* variants occurring in approximately 50% and *PIK3CA* variants in approximately 36% of clear cell cases.^9^

Mucinous ovarian cancer (MOC) comprises approximately 3% of EOC.^21^ Often heterogeneous, a single tumour may comprise different tissues including benign, borderline and invasive elements.^17^ The genetic abnormalities differ from EOC, with nearly 100% harbouring a pathogenic somatic variant in *KRAS* and high frequency of *ERBB2* amplification.^6^ MOC shares many of its molecular biological characteristics with gastrointestinal tumours, and is differentiated from HGSOC and colorectal cancer through immunohistochemical staining for CK7 and CK20.^21^ The understanding of MOC is now at the point where it is considered a separate disease entity to other EOCs.^21^

### 1.2 Risk factors

One of the most relevant risk factors for EOC is a family history of breast and/or ovarian cancer (HBOC). Traditionally treated the same in clinical and research settings (although differences in terms of molecular and clinical characteristics have been noted) EOC and primary peritoneal cancer (PPC) are thought to be similarly hereditary and have similar family histories of breast and/or ovarian cancer.^22^ There is a 3‐fold increase in risk of developing ovarian cancer in women with a first‐degree relative with ovarian cancer.^23^ The relative risk (RR) is higher for first‐degree relatives diagnosed <50 years than for those >50 (4.7 vs 2.5, *P* = .0052). Having serous ovarian cancer carries with it a higher RR for first‐degree relatives than non‐serous ovarian cancer (RR = 3.6 vs 2.3, *P* = .023).^24^

Hormonal and reproductive factors are the most significant other risk factors. A higher lifetime number of menstrual cycles is associated with a higher risk of EOC,^25^ suggesting that ovulation is involved in ovarian carcinogenesis. Factors that reduce ovulation, including pregnancy, breastfeeding and the oral contraceptive pill, are protective and nulliparity associated with higher risk.^26^, ^27^, ^28^ Hormone replacement therapy (HRT) carries a modest but persistent risk,^29^ as do increased height, weight and body mass index.^30^, ^31^ There is no significant association with diet or alcohol.^32^, ^33^, ^34^ Tobacco smoking is associated only with MOC.^35^ Endometriosis is associated with 15% to 20% of clear cell and endometrioid ovarian cancer, and carries up to a 3‐fold risk.^36^, ^37^, ^38^

### 1.3 Epithelial ovarian cancer susceptibility genes

Frequencies of pathogenic variants in high, moderate and low penetrance (commonly defined as ≥10%, 5%‐9% and ≤ 4%) EOC susceptibility genes in the unselected ovarian cancer population and HBOC families vary with population number, characteristics, geography, cancer subtype and technique used in analysis. These frequencies are summarized in Table S1, cancer‐associated risks in Table S2 and comparisons between frequency and risk between the general population, unselected EOC and HBOC families in Table 1.

**Table 1:** Comparing frequency of EOC susceptibility genes in different populations and cumulative lifetime risk of ovarian cancer

Gene	Frequency in families with ≥3 EOC (%)^a^	Frequency in HBOC families (%)	Frequency in unselected EOC cases (%)	Frequency in general population (%)	Cumulative lifetime risk (%)
BRCA1	60	3.7‐25.0^102^ ^,135^	3.8‐15.5^51^, ^71^, ^93^	0.2‐0.3^47^ ^,136,137^	13‐60^93^, ^102^
BRCA2	20	3.9‐13.0^102^ ^,135^	3.4‐5.5^51^, ^71^, ^93^	0.2‐0.3^47^ ^,136,137^	10‐25^93^, ^102^
RAD51C	0	0.5‐0.8^11^, ^102^	0.32‐2.5^11^, ^57^	0.002^57^	5‐11^11^, ^57^
RAD51D	0	0.88^12^	0.3‐0.6^57^, ^59^	0.002^57^	10‐12^12^, ^57^
BRIP1	0	0.5‐1.71^70^, ^102^	0.4‐1.4^59^, ^71^	0.002^13^	5.8^13^
PALB2	0	0.21‐0.9^70^, ^102^	0.4‐1.1^71,137^	0.13^138^	NC
BARD1	0	2.75^139^	0.14‐0.21^59^ ^,137^	0.13^138^	NC
CHEK2	0	0.43‐1.1^70^, ^102^	0.4‐0.57^59^, ^71^	0.97^138^	NC
ATM	0	0.65‐2.59^103^ ^,140^	0.45‐0.87^71^ ^,137^	0.38^138^	NC
NBN	0	0.21‐0.32^70^, ^103^	0.38‐0.47^59^ ^,137^	0.17^138^	NC
TP53	0	0.16‐0.5^102^, ^103^	0.31^59^	0.07^138^	NC
MMR genes	0	<0.3‐1.72^70^, ^102^	0.4‐0.6^59^, ^71^	0.51^138^	4‐12^84^, ^93^
MSH2	0	NC	0.38–0.4^71^ ^,137^	0.03^138^	6‐24^88^ ^,141^
MLH1	0	NC	0.05‐0.10^59^, ^93^	0.05^138^	6‐20^88^ ^,141^
MSH6	0	1.29^70^	0.16‐0.65^59^ ^,137^	0.13^138^	1^141^
PMS2	0	NC	0.2‐0.43^59^ ^,137^	0.29^138^	NC

a Based on 34 families with three or more proven EOC in Manchester with testing ovarian probands.

EOC, epithelial ovarian cancer; HBOC, history of breast and/or ovarian cancer; NC, not calculated.

### 1.4 Homologous recombination genes

Many of the proteins and related genes involved in homologous recombination (HR) have been associated with risk of ovarian cancer, due to the significant role HR has been shown to play in ovarian carcinogenesis. The Cancer Genome Atlas (TCGA) found HR to be defective in approximately half of 489 women with stage II to IV HGSOCs,^10^ attributed to germline variants in *BRCA1* (in 9% of tumours) or *BRCA2* (8%), somatic variants in *BRCA1* or *BRCA2* (3%), epigenetic silencing of *BRCA1* (11%), amplification of *EMSY* (8%), *PTEN* deletion/mutation (7%), hypermethylation of *RAD51C* (3%), *ATM* or *ATR* pathogenic variants (2%) and variants of other HR genes (5%).^10^, ^39^, ^40^ However, TCGA did not find any germline variants in likely significant genes *RAD51C* or *RAD51D*, and have been criticized for inaccurate results due to technical artefacts, particularly affecting the ovarian cancer cases.^41^ Homologous recombination deficient (HRD) ovarian cancers have greater sensitivity to DNA‐damaging agents that crosslink DNA such as cisplatin as HR is required for the repair of these lesions, and improved OS.^36^, ^42^, ^43^ Being able to identify women with HRD cancers has clear clinical implications in terms of chemotherapy regime planning and development and use of targeted therapies.

### 1.5 BRCA genes, BRCAness and methylation

Identified in 1990 and mapped to chromosome 17q21, *BRCA1* plays essential roles in DNA damage repair, cell‐cycle arrest, transcriptional activation, chromatin remodelling, apoptosis and genetic stability.^40^, ^44^ In cancer patients, pathogenic *BRCA1* variants most commonly occur in areas that are important in subcellular localization and interaction with partner proteins (the N‐terminal RING domain encoded by exons 2 to 7, coding regions of exons 11 to 13, and BRCA1 C‐terminus encoded by the BRCT domain or exons 16 to 24).^44^ Frequencies of epigenetic/genetic mechanisms of *BRCA1* aberration have been noted to vary between ethnicity, with pathogenic variants predominating in White Europeans, and methylation in people of African descent.^45^ Pathogenic variants in *BRCA1* are the most highly penetrant EOC susceptibility genes. A first‐degree relative of a woman with *BRCA1*‐related EOC has a RR of 21.0 (95% CI 11.9‐36.8)^24^ of developing EOC herself and affected women develop predominantly serous ovarian cancer approximately a decade earlier than average.^46^, ^47^

In 1994, *BRCA2* was localized to 13q12‐q13.^48^ While there are some similarities there is no significant sequence homology between *BRCA1* and *BRCA2* exon structures. It is a transcriptional co‐regulator involved in DNA recombination and repair processes, in particular regulation of *RAD51* and maintenance of genomic stability.^40^, ^44^ A sequence called the BRC motif is the major domain for RAD51 interactions.^44^ Affected women develop cancer 3 to 6 years earlier than average. The RR of ovarian cancer to a first‐degree relative is estimated to be 9.6 (95% CI 5.3‐17.5).^24^

Approximately 0.2% to 0.5% of women carry a pathogenic *BRCA* variant.^49^, ^50^ This varies by population; in Ashkenazi Jewish women up to 2.5% have a pathogenic *BRCA* variant and 29% to 41% of ovarian cancer is attributed to one of three *BRCA* founder variants (c.68_69delAG and c.5266dupC in *BRCA1* and c.5946delT in *BRCA2*) compared to 10% in the overall outbred ovarian cancer population.^51^ In Iceland, the *BRCA2* variant c.999del5 carries an odds ratio (OR) of 20.7 and accounts for 6.0% to 7.9% of ovarian cancer in that country.^52^

Germline *BRCA1* or *BRCA2* variants were reported by Alsop et al in approximately 15% of ovarian cancer patients, and approximately 23% of patients with HGSOC.^53^ Overall, 25.4% of the observed RR in first‐degree relatives is thought to be accounted for by *BRCA1* and *BRCA2* variants.^24^ Since the discovery of *BRCA1* and *BRCA2*, the phenotype “BRCAness”: patients with genomic instability, serous histology, high response rates to platinum‐based chemotherapy, long treatment‐free intervals, good OS but without a detected *BRCA* variant, has been described.^43^, ^54^ Attempts to identify BRCAness more distinctly with molecular classification are ongoing.^40^ Being able to identify this patient group reliably could allow management to be tailored in a more targeted manner and allow greater a number of patients to access treatments currently restricted to those with a *BRCA* variant.

Epigenetic mechanisms of *BRCA* inactivation such as promoter methylation causing transcriptional silencing of cancer‐associated genes have also been identified.^40^, ^55^ Methylation in cancer has been found to occur in the cytosine residues in CpG dinucleotides which occur in the promoters of many genes. Up to one‐third of ovarian cancers show dysfunctional methylation of the *BRCA1* promoter^40^ to the extent that in most cases *BRCA1* expression is undetectable. An example is two HBOC families recently described to have a dominantly inherited 5’ UTR variant (c.‐107 T > A), associated with epigenetic *BRCA1* silencing caused by promoter hypermethylation.^56^ The clinical features of the affected women were consistent with the *BRCA1* phenotype.

#### 1.5.1 Other homologous recombination genes

The gene, *RAD51C*, isolated in 1998 and localized to chromosome 17q23,^57^ is one of the five RAD51 paralogs. Together, their protein products form the BCDX2 complex responsible for RAD51 recruitment and stabilization at DNA damage sites.^58^ Pathogenic variants have been found in a functional domain in the C‐terminus of the protein, an area important in forming RAD51B‐RAD51C‐RAD51D‐XRCC2 and RAD51C‐XRCC2 complexes, and therefore, in double‐strand DNA repair, demonstrating the influence of variants on HR.^59^ Affected women may develop EOC up to 6 years earlier than the general population.^58^, ^60^, ^61^ The risk is higher for serous ovarian cancer (OR = 7.4, 95% CI 1.6‐35.0) compared to all ovarian cancer subtypes (OR = 5.2, 95% CI 1.1‐24.0, *P* = .035) and the cumulative lifetime risk is 5% to 11%.^11^, ^59^

Its paralog *RAD51D,* also isolated in 1998, is localized to 17q11.^62^ It recruits RAD51 to DNA damage sites and is vital during embryonic development.^63^, ^64^ Pathogenic variants have been found in the C‐terminal region involved in binding to RAD51C.^59^ Short interfering RNAi reagents targeting *RAD51D* have been observed to cause sensitivity to the PARP inhibitor, olaparib, similar to that seen by *BRCA2* silencing.^12^ This suggests that PARP inhibitors could be used in patients with *RAD51D* variants. Variants most commonly occur in HGSOC^59^ and are estimated to confer a 6‐fold increase in ovarian cancer risk, equating to approximately 10% cumulative risk by age 80.^12^ Affected women may develop EOC up to 9 years earlier than the general population.^61^

The gene *PALB2*, was discovered in 2006. PALB2 protein localizes with BRCA2 in nuclear foci, promoting localization, stability and enabling recombinational repair and checkpoint functions.^65^ It also directly affects RAD51 function, promoting RAD51‐mediated D‐loop formation and DNA binding.^66^ Although early studies did not confirm a significant increase in EOC risk it now looks likely that this was due to studies being underpowered, and cumulative lifetime risk of EOC is not known. Affected women may develop EOC up to 7 years earlier than the general population.^67^

The protein BRIP1 interacts with BRCA1 through BRCT repeats at the c‐terminal end of BRCA1 and is required for normal repair of double‐strand DNA breaks.^68^ Pathogenic variants in *BRIP1* are predicted to truncate the protein before this BRCA1 binding domain.^13^ Pathogenic variants in *BRIP1* increase cell sensitivity to DNA‐crosslinking agents^69^ making patients with these variants more likely to be sensitive to platinum‐based chemotherapy. An Icelandic study found the frameshift deletion c.2040_204insTT to be associated with increased risk of ovarian cancer (OR = 8.1, 95% CI 4.7‐13.9, *P* = 2.8 x 10^−14^) and average four years poorer OS.^70^ Pathogenic variant carriers develop EOC at the same age as in the general population, and have an estimated 5.8% cumulative lifetime risk.^13^, ^67^

Other low‐risk HR genes for which only weak or insignificant associations have been found with EOC include ataxia telangiectasia (ATM), checkpoint kinase 2 (*CHEK2*) and nibrin (*NBN*)^13^, ^61^, ^71^, ^72^, ^73^, ^74^, ^75^

### 1.6 Mismatch repair genes

The mismatch repair (MMR) system involves seven genes: *MLH1*, *MSH2*, *MSH6*, *PMS2, PMS1*, *MSH3* and MLH3.^76^ However, only the first four genes are clearly associated with increased cancer risk when pathogenic variants are inherited. Dysfunction of MMR can result from epigenetic and genetic mechanisms, and the responsible germline variants in ovarian cancer are described most frequently in *MSH6* 76, 77, 78 Loss of MMR function and subsequent microsatellite instability (MSI) is associated with Lynch syndrome (LS). The cumulative lifetime risk of ovarian cancer in women with LS has been estimated at 4% to 12%,^76^ although in our centre we found a cumulative risk of 20%.^79^ Affected women can develop ovarian cancer in their 40s, 15 to 22 years earlier than the general population.^76^ Women with truncating pathogenic variations have been observed to be older (median 6.3 years) at diagnosis.^80^ Analysis of contribution of individual MMR genes has found significant cumulative lifetime risks of ovarian cancer for *MSH2* and *MLH1* pathogenic variant carriers (6%‐24%)^81^, ^82^ and *MSH6* carriers (1%‐13%).^82^, ^83^

The prevalence of MMR‐deficiency or microsatellite instability (MSI) in familial ovarian cancer has been estimated between 10% and 20%.^76^, ^84^ Loss of MMR expression is more commonly found in non‐serous ovarian cancer, particularly endometrioid and clear cell carcinomas.^85^ Mean age at diagnosis in women with pathogenic germline MMR variants is 9 to 13 years earlier than the general population and cumulative lifetime risk of ovarian cancer has been reported as low as 3.7% (1.4%‐13%).^86^ Prognosis is affected by MMR variants. PFS is longer for MMR‐deficient women compared to MMR‐low and MMR‐proficient ovarian cancer (*P* = .0046). They are also more likely to be diagnosed at an earlier stage (*P* = .0041).^87^ Ten‐year ovarian cancer‐specific survival has been found to be 80.6% in one series of MMR pathogenic variant carriers with ovarian cancer.^88^ High mRNA expression of *MSH6*, *MLH1*, and *PMS2* is associated with a significantly improved OS.^89^ It has been suggested these patients could be good candidates for checkpoint inhibitors.^87^

#### 1.6.1 TP53

The crucial role of *TP53* is exemplified by Li‐Fraumeni syndrome, a disorder with close to 100% cancer incidence by age 70^90^; the median age of ovarian cancer in these patients is 39.5 years.^91^ Variants have been associated with ovarian cancer risk (OR = 18.50, 95% CI 2.56‐808.1).^92^ However, numerous studies have not found germline *TP53* variants to be significantly associated with ovarian cancer or to affect risk.^61^, ^93^, ^94^, ^95^, ^96^

### 1.7 Other syndromic associations

A number of other syndromic associations with ovarian cancer have been reported, such as with Peutz‐Jeghers disease, although this association is not with EOC.^97^ Another probably false association that has been frequently quoted is with Gorlin syndrome, an autosomal dominant condition associated with increased risk of childhood‐onset brain tumours.^98^ The latter may well be linked to transformation of benign ovarian fibromas to fibrosarcoma due to childhood spinal irradiation to treat medulloblastoma.^99^

### 1.8 Interventions for women carrying a pathogenic variant

When a pathogenic variant is identified, it is essential that affected women are offered risk‐reduction interventions and cascade testing be offered to relatives. Uptake of cascade testing in this situation has been noted to be relatively low, estimated at 15% to 57% in one systematic review^100^ and genetic testing in eligible women with ovarian cancer also low. The reasons for this are likely multifactorial, including insufficient referrals to clinical genetics, variable reporting of relatives by probands, inadequate understanding and communication of tests, feelings of irrelevance and deferring the process by relatives.^100^, ^101^, ^102^ The use of screening has been investigated. The risk of ovarian cancer algorithm (ROCA) using serum CA125 and transvaginal ultrasound has been proposed for high‐risk women; however, impact on survival is not known.^103^

A meta‐analysis found an 80% reduction in ovarian/fallopian tube cancer associated with risk‐reducing salpingo‐oophorectomy (RRSO), with greater risk reduction likely in *BRCA2* carriers than *BRCA1* carriers.^104^ RRSO is recommended for pre‐menopausal women with pathogenic *BRCA1*/2 variants who have completed childbearing.^105^, ^106^ The National Comprehensive Cancer Network (NCCN) recommends offering RRSO to *RAD51C, RAD51D, BRIP1* and *BRCA2* carriers at age 45 to 50 and 35 to 40 for *BRCA1* carriers.^106^ While other studies have found risk reduction of breast cancer following RRSO of approximately 50%, these studies have been criticized for heavy bias,^107^ and a subsequent study using methodology to minimize bias found no evidence of a protective effect (HR 1.09 [95% CI 0.67‐1.77]).^108^ While tubal ligation has been shown to reduce risk of non‐mucinous serous EOC^109^ and studies on the role of risk‐reducing bilateral salpingectomy have shown some benefit^110^ there is currently insufficient evidence for these procedures to be recommended by clinical guidelines.^3^, ^111^ However, opportunistic salpingectomy is recommended at time of hysterectomy for benign conditions in the general population.^112^

PARP inhibitors demonstrate synthetic lethality in HR‐defective cells.^66^ They have been successfully investigated preclinically,^113^, ^114^ in phases I, II and III trials with olaparib,^115^, ^116^, ^117^, ^118^, ^119^, ^120^, ^121^, ^122^, ^123^ niraparib^124^ and rucaparib.^125^ The FDA‐granted approval of olaparib for patients with *BRCA1*/2‐associated advanced ovarian cancer after ≥3 lines of chemotherapy in 2014.^126^ Most recently, the SOLO1 trial (NCT01844986) has demonstrated significant benefit after first‐line platinum‐based chemotherapy with olaparib compared to placebo (HR = 0.30, 95% CI 0.23‐0.41, *P* < .001) suggesting PARP inhibitors can be utilized clinically earlier in treatment plans.^127^

### 1.9 Single nucleotide polymorphisms

In addition to high and moderate penetrance susceptibility genes, multiple common but low penetrance susceptibility alleles have been identified by candidate gene studies and genome‐wide association studies (GWAS). At least 34 susceptibility loci for different EOC subtypes have been identified to date, of which 27, associated with invasive EOC, account for approximately 6.4% of the population's polygenic risk.^128^ These loci are listed in Table S3. These GWAS have also identified SNPs associated with reduced risk of ovarian cancer.

### 1.10 Risk models and polygenic risk scores

Perhaps the most useful model to assess ovarian cancer cumulative risk is the BOADICEA model (https://pluto.srl.cam.ac.uk/cgi-bin/bd4/v4beta14/bd.cgi).^129^ This combines family history of breast and ovarian cancer to assess breast and ovarian cancer risk. It is being adapted to include non‐genetic risks such as reproductive and hormonal factors as well as the more recently identified genes such as *PALB2* in addition to *BRCA1/2*. The addition of an SNP polygenic risk score (PRS) is also anticipated.

The combination of genetic information from GWAS and lifestyle/reproductive factors have been used to create polygenic risk scores. In breast cancer, PRSs have been used to detail an individual woman's risk more accurately. A study using 77 breast cancer‐associated SNPs showed women in the highest 1% had a 3‐fold increase in breast cancer risk compared to the middle quintile (OR 3.36, 95% CI 2.95‐3.83).^130^ The PRS has been further developed in combination with non‐genetic risk factors and mammographic breast density.^131^

The question of whether a polygenic score can be applied to ovarian cancer was speculatively addressed by Jervis et al in 2014 using an 11‐SNP panel.^24^ The familial RR increased with increasing PRS; however, this was not statistically significant. The RRs for relatives of probands in the highest quartile (RR 2.61, 95% CI 1.61‐4.24) were also estimated to be lower than for those in the 25th to 75th quartiles (RR 3.83, 95% CI 2.56‐5.73 for 50th‐75th quartile). It was proposed that this was due to the small number of SNPs used.

There are limitations to PRSs and currently there is no consensus among clinicians of their utility. Models use varying SNPs, not always including the most significant germline pathogenic variants, and GWAS often include individuals from European ancestry, limiting the predictive ability of a PRS in non‐European ancestry women.

## 2 CONCLUSION

The heritability of ovarian cancer has not been completely explained. Pathogenic variants in moderate‐to‐high risk genes such as *BRCA1* and *BRCA2*, *RAD51C*/*D* and those involved in mismatch repair contribute to approximately 20% to 25% of all epithelial ovarian cancers,^24^, ^132^ and GWAS‐identified variants have been estimated to account for approximately 6.4% of polygenic ovarian cancer risk.^133^ However, a significant proportion of women who develop ovarian cancer with a strong family history of breast and/or ovarian cancer still do not have a known variant to explain their increased risk, and there must be other genetic factors at play that we do not yet understand. A crucial question is also at what point women undergo genetic testing. Given the detection rate of HR‐related pathogenic variants including *BRCA1/2* in EOC patients is well above 10%, an argument has been made that women should have genetic testing on the basis of ovarian pathology alone.^134^

We also need to understand further the precise risks attributable to the genetic and lifestyle factors that have already been identified. The confidence intervals of the level of risk attributable to the known genetic variants are wide. Greater precision is needed to improve provision of information about specific risks to individuals with a family history of ovarian cancer, or known genetic risk factors, and how this affects their family. Making decisions regarding family planning and risk reduction strategies can be stressful for patients. Physicians, surgeons, and the clinical genetics team need to be able to communicate these complex risk‐association issues as accurately as possible to provide the best support for their patients.

## DATA ACCESSIBILITY

All data generated or analysed for this review are included in this published article and supplementary files.

## Supporting information


**TABLE S1** Frequency of variants in epithelial ovarian cancer susceptibility genes in ovarian cancer patients

**TABLE S2** Risk estimates of ovarian cancer associated with epithelial ovarian cancer susceptibility genes

**TABLE S3** Single nucleotide polymorphisms identified in genome‐wide association studies associated with epithelial ovarian cancer risk
