# Supplementary material for: Epithelial ovarian cancer risk: A review of the current genetic landscape
Source: Clin Genet. 2019 May 29;97(1):54–63. doi: 10.1111/cge.13566 (PMC7017781; doi:10.1111/cge.13566)
Supplement: Supplementary file 1 — TABLE S1 Frequency of variants in epithelial ovarian cancer susceptibility genes in ovarian cancer patients [file CGE-97-54-s001.docx]

### Supplementary Table 1: Frequency of variants in EOC susceptibility genes in ovarian cancer patients

| Gene | Lead Author | Year | Number of cases | Frequency in unselected  OC cases (%) | Frequency in HBOC cases (%) |
| --- | --- | --- | --- | --- | --- |
| BRCA1 | Evans et al ^135^ | 2008 | 442 |  | 25.0 |
|  | Zhang et al ^136^ | 2011 | 1342 | 8.1 |  |
|  | Alsop et al ^51^ | 2012 | 1001 | 8.8 |  |
|  | Song et al ^93^ | 2014 | 2222 | 3.8 |  |
|  | Norquist et al ^59^ | 2016 | 1915 | 9.5 |  |
|  | Harter et al ^71^ | 2017 | 523 | 15.5 |  |
|  | Lilyquist et al ^137^ | 2017 | 7768 | 4.0 |  |
|  | Castera et al ^102^ | 2018 | 4409 |  | 3.7 |
| BRCA2 | Evans et al ^135^ | 2008 | 442 |  | 13.0 |
|  | Zhang et al ^136^ | 2011 | 1342 | 5.1 |  |
|  | Alsop et al ^51^ | 2012 | 1001 | 5.3 |  |
|  | Song et al ^93^ | 2014 | 2222 | 4.2 |  |
|  | Norquist et al ^59^ | 2016 | 1915 | 5.1 |  |
|  | Harter et al ^71^ | 2017 | 523 | 5.5 |  |
|  | Lilyquist et al ^137^ | 2017 | 7768 | 3.4 |  |
|  | Castera et al ^102^ | 2018 | 4409 |  | 3.9 |
| RAD51C | Meindl et al ^58^ | 2010 | 1100 |  | 0.55 |
|  | Loveday et al ^11^ | 2012 | 272 | 1.1 |  |
|  | Loveday et al ^11^ | 2012 | 1132 |  | 0.80 |
|  | Blanco et al ^56^ | 2014 | 516 |  | 0.6 |
|  | Minion et al† ^70^ | 2015 | 466 |  | 0.64 |
|  | Song et al ^57^ | 2015 | 3429 | 0.32 |  |
|  | Norquist et al ^59^ | 2016 | 1915 | 0.6 |  |
|  | Harter et al ^71^ | 2017 | 523 | 2.5 |  |
|  | Lilyquist et al ^137^ | 2017 | 7768 | 0.8 |  |
|  | Castera et al ^102^ | 2018 | 4409 |  | 0.5 |
| RAD51D | Loveday et al ^12^ | 2011 | 911 |  | 0.88 |
|  | Song et al ^57^ | 2015 | 3429 | 0.35 |  |
|  | Norquist et al ^59^ | 2016 | 1915 | 0.6 |  |
|  | Harter et al ^71^ | 2017 | 523 | 0.6 |  |
|  | Lilyquist et al ^137^ | 2017 | 7768 | 0.3 |  |
| BRIP1 | Minion et al† ^70^ | 2015 | 466 |  | 1.71 |
|  | Norquist et al ^59^ | 2016 | 1915 | 1.4 |  |
|  | Harter et al ^71^ | 2017 | 523 | 0.4 |  |
|  | Lilyquist et al ^137^ | 2017 | 7768 | 1.0 |  |
|  | Castera et al ^102^ | 2018 | 4409 |  | 0.5 |
| PALB2 | Minion et al† ^70^ | 2015 | 466 |  | 0.21 |
|  | Schroeder et al ^103^ | 2015 | 620 |  | 0.32 |
|  | Norquist et al ^59^ | 2016 | 1915 | 0.6 |  |
|  | Harter et al ^71^ | 2017 | 523 | 1.1 |  |
|  | Lilyquist et al ^137^ | 2017 | 7768 | 0.4 |  |
|  | Castera et al ^102^ | 2018 | 4409 |  | 0.9 |
| BARD1 | Ratajska et al ^138^ | 2012 | 109 |  | 2.75 |
|  | Norquist et al ^59^ | 2016 | 1915 | 0.21 |  |
|  | Lilyquist et al ^137^ | 2017 | 7768 | 0.14 |  |
| CHEK2 | Schroeder et al ^103^ | 2015 | 620 |  | 0.97 |
|  | Minion et al† ^70^ | 2015 | 466 |  | 0.43 |
|  | Norquist et al ^59^ | 2016 | 1915 | 0.57 |  |
|  | Harter et al ^71^ | 2017 | 523 | 0.4 |  |
|  | Lilyquist et al ^137^ | 2017 | 7768 | 0.43 |  |
|  | Castera et al ^102^ | 2018 | 4409 |  | 1.1 |
| ATM | Thorstenson et al ^139^ | 2003 | 270 |  | 2.59 |
|  | Schroeder et al ^103^ | 2015 | 620 |  | 0.65 |
|  | Minion et al† ^70^ | 2015 | 466 |  | 0.86 |
|  | Norquist et al ^59^ | 2016 | 1915 | 0.57 |  |
|  | Harter et al ^71^ | 2017 | 523 | 0.45 |  |
|  | Lilyquist et al ^137^ | 2017 | 7768 | 0.87 |  |
|  | Tavera-Tapia et al† ^140^ | 2017 | 1477 |  | 1.78 |
|  | Castera et al ^102^ | 2018 | 4409 |  | 1.0 |
| NBN | Schroeder et al ^103^ | 2015 | 620 |  | 0.32 |
|  | Minion et al^ ^70^ | 2015 | 466 |  | 0.21 |
|  | Norquist et al ^59^ | 2016 | 1915 | 0.47 |  |
|  | Harter et al ^71^ | 2017 | 523 | 0.4 |  |
|  | Lilyquist et al ^137^ | 2017 | 7768 | 0.38 |  |
| TP53 | Schroeder et al ^103^ | 2015 | 620 |  | 0.16 |
|  | Norquist et al ^59^ | 2016 | 1915 | 0.31 |  |
|  | Castera et al ^102^ | 2018 | 4409 |  | 0.5 |
| MMR  MSH6  MSH2  MLH1  PMS2 | Song et al ^93^ | 2014 | 2222 | 0.77  0.45  0.2  0.1  0.05 |  |
| MMR  MSH6 | Minion et al† ^70^ | 2015 | 466 |  | 1.72  1.29 |
| MMR  PMS2  MSH6  MLH1 | Norquist et al ^59^ | 2016 | 1915 | 0.4  0.2  0.16  0.05 |  |
| MMR  MSH2  MSH6 | Harter et al ^71^ | 2017 | 523 | 0.6  0.4  0.2 |  |
| MSH6  MSH2  PMS2  MLH1 | Lilyquist et al ^137^ | 2017 | 7768 | 0.65  0.38  0.43  0.08 |  |
| MMR | Castera et al ^102^ | 2018 | 4409 |  | <0.3 |

† = studies included only BRCA-negative patients

HBOC = Hereditary breast and/or ovarian cancer

OC = Ovarian cancer
