# Supplementary material for: Epithelial ovarian cancer risk: A review of the current genetic landscape
Source: Clin Genet. 2019 May 29;97(1):54–63. doi: 10.1111/cge.13566 (PMC7017781; doi:10.1111/cge.13566)
Supplement: Supplementary file 2 — TABLE S2 Risk estimates of ovarian cancer associated with epithelial ovarian cancer susceptibility genes [file CGE-97-54-s002.docx]

### Supplementary Table 2: Risk estimates of ovarian cancer associated with EOC susceptibility genes

| Gene | Authors | Year | Number of cases | Relative Risk  (95% CI) | P Value | Odds Ratio  (95% CI) | P Value |
| --- | --- | --- | --- | --- | --- | --- | --- |
| BRCA1 | Song et al ^93^ | 2014 | 2222 | - | - | 60 (10-2,100) | - |
|  | Norquist et al ^59^ † | 2016 | 1915 | - | - | 48.9 (24.0-100)  29.0 (22.7-37.1) | <0.001  <0.001 |
|  | Lilyquist et al ^137^ | 2017 | 7768 | 11.78 (10.42-13.28) | 3.5x10^-182^ | - | - |
|  | Castera et al  (HBOC) ^102^ | 2018 | 5131 | - | - | 13.22 (10.01-17.22) | - |
| BRCA2 | Song et al ^93^ | 2014 | 2222 | - | - | 17 (6.3-63) | - |
|  | Norquist et al ^59^ † | 2016 | 1915 | - | - | 14.0 (8.2-23.8)  12.7 (9.7-16.4) | <0.001  <0.001 |
|  | Lilyquist et al ^137^ | 2017 | 7768 | 7.96 (7.00-9.01) | 2.3x10^-99^ | - | - |
|  | Castera et al (HBOC) ^102^ | 2018 | 5131 | - | - | 8.61 (6.78-10.86) | - |
| RAD51C | Loveday et al ^11^ | 2012 | 1404 | 5.88 (2.91-11.88) | 7.65x10^-7^ | - | - |
|  | Song et al ^57^ | 2015 | 3429 | - | - | 5.2 (1.1-24) | 0.035 |
|  | Norquist et al ^59^ † | 2016 | 1915 | - | - | 15.8 (1.9-128)  3.4 (1.5-7.6) | 0.002  0.005 |
|  | Lilyquist et al ^137^ | 2017 | 7768 | 5.12 (3.72-6.88) | 1.1x10^-16^ | - | - |
|  | Castera et al (HBOC) ^102^ | 2018 | 5131 | - | - | 4.54 (2.55-7.48) | - |
|  | Lu et al ^101^ | 2018 | 2051 | - | - | - | 0.004 |
| RAD51D | Loveday et al ^12^ | 2011 | 911 | 6.30 (2.86-13.85) | 4.8x10^-6^ | - | - |
|  | Song et al ^57^ | 2015 | 3429 | - | - | 12 (1.5-90) | 0.10 |
|  | Norquist et al ^59^ † | 2016 | 1915 | - | - | 9.0 (1.9-42.5)  10.9 (4.6-26.0) | 0.002  <0.001 |
|  | Lilyquist et al ^137^ | 2017 | 7768 | 6.34 (3.16-11.34) | 4.4x10^-6^ | - | - |
|  | Castera et al (HBOC) ^102^ | 2018 | 5131 | - | - | 5.23 (1.46-13.17) | - |
| BRIP1 | Norquist et al ^59^ † | 2016 | 1915 | - | - | 9.1 (3.4-24.2)  6.4 (3.8-10.6) | <0.001  <0.001 |
|  | Lilyquist et al ^137^ | 2017 | 7768 | 4.99 (3.79-6.45) | 2.9x10^-21^ | - | - |
|  | Castera et al (HBOC) ^102^ | 2018 | 5131 |  |  | 2.49 (1.42-3.97) |  |
| PALB2 | Norquist et al ^59^ † | 2016 | 1915 | - | - | 10.2 (2.2-47.0)  4.4 (2.1-9.1) | <0.001  <0.001 |
|  | Lilyquist et al ^137^ | 2017 | 7768 | 3.08 (1.93-4.67) | 1.2x10^-5^ | - | - |
|  | Castera et al (HBOC) ^102^ | 2018 | 5131 | - | - | 8.22 (4.91-13.05) | - |
| BARD1 | Norquist et al ^59^ † | 2016 | 1915 | - | - | 20.3 (1.1-377)  4.2 (1.4-12.5) | 0.009  0.02 |
|  | Lilyquist et al ^137^ | 2017 | 7768 | 1.28 (0.55-2.51) | 0.59 | - | - |
| CHEK2 | Norquist et al ^59^ † | 2016 | 1915 | - | - | 0.6 (0.3-1.5)  0.4 (0.2-0.9) | 0.37  0.04 |
|  | Lilyquist et al ^137^ | 2017 | 7768 | 0.98 (0.75-1.27) | 0.87 | - | - |
|  | Castera et al (HBOC) ^102^ | 2018 | 5131 | - | - | 1.67 (1.18-2.27) | - |
| ATM | Norquist et al ^59^ † | 2016 | 1915 | - | - | 2.5 (1.0-6.2)  2.4 (1.2-4.7) | 0.07  0.01 |
|  | Lilyquist et al ^137^ | 2017 | 7768 | 2.25 (1.69-2.94) | 1.8x10^-7^ | - | - |
|  | Castera et al (HBOC) ^102^ | 2018 | 5131 | - | - | 3.20 (2.14-4.53) | - |
|  | Lu et al ^101^ | 2018 | 2051 | - | - | 2.85 (1.30-6.32) |  |
| NBN | Norquist et al ^59^ † | 2016 | 1915 | - | - | 2.2 (0.7-7.0)  2.3 (0.99-5.4) | 0.26  0.09 |
|  | Lilyquist et al ^137^ | 2017 | 7768 | 2.03 (1.27-3.08) | 0.004 | - | - |
| TP53 | Norquist et al ^59^ † | 2016 | 1915 | - | - | 3.4 (0.95-12.0)  2.9 (1.2-6.9) | 0.08  0.03 |
|  | Castera et al (HBOC) ^102^ | 2018 | 5131 | - | - | 1.56 (0.85-2.52) | - |
|  | Lu et al ^101^ | 2018 | 2051 | - | - | 18.5 (2.56-808.1) | - |
| MMR | Song et al ^93^ | 2014 | 2222 | - | - | 2.3 (0.83-8.2) | - |
| MLH1  MSH2  MSH6  PMS2 | Lilyquist et al ^137^ | 2017 | 7768 | 2.20 (0.81-4.78)  13.91 (8.82-20.87)  5.04 (3.70-6.70)  1.48 (0.81-2.48) | 0.12  2.5x10^-17^  1.7x10^-17^  0.2 | - | - |
| MSH6 | Castera et al (HBOC) ^102^ | 2018 | 5131 | - | - | 2.50 (1.12-4.67) | - |
| MSH6 | Lu et al ^101^ | 2018 | 2051 | - | - | 4.16 (1.95-9.47) | - |

† = OR on top line of cases vs NHLBI Exome Sequencing Project European American dataset (ESP EA) (n=4300), OR on lower line of cases vs the Exome Aggregation Consortium (ExAC) (n=36,276).

HBOC: Hereditary breast and/or ovarian cancer
