# Supplementary material for: Epithelial ovarian cancer risk: A review of the current genetic landscape
Source: Clin Genet. 2019 May 29;97(1):54–63. doi: 10.1111/cge.13566 (PMC7017781; doi:10.1111/cge.13566)
Supplement: Supplementary file 3 — TABLE S3 Single nucleotide polymorphisms identified in genome‐wide association studies associated with epithelial ovarian cancer risk [file CGE-97-54-s003.docx]

### Supplementary Table 3: Table of SNPs identified in GWAS associated with EOC risk

| SNP | Location | Closest Gene | Per-allele OR (95% CI) | P-value | Reference |
| --- | --- | --- | --- | --- | --- |
| rs3814113  ^†^  ^‡^  Others in LD | 9p22.2 | BNC2, CNTLN, LOC648570 | 0.79 (0.75-0.84)  0.77 (0.73-0.81)  0.86 (0.79-0.94) | 2.47x10^-17^  4.1x10^-21^  0.001 | Song (OCAC) ^145^ |
| rs4445329 | 9p22.2 | “ | 0.79 (0.75-0.84) | 2.67x10^-17^ | Song (OCAC) ^145^ |
| rs10810666 | 9p22.2 | “ | 0.80 (0.75-0.85) | 1.24x10^-12^ | Song (OCAC) ^145^ |
| rs10962656 | 9p22.2 | “ | 0.81 (0.76-0.87) | 5.88x10^-9^ | Song (OCAC) ^145^ |
| rs12379183 | 9p22.2 | “ | 0.82 (0.78-0.87) | 1.36x10^-10^ | Song (OCAC) ^145^ |
| rs2153271 | 9p22.2 | “ | 0.85 (0.81-0.90) | 4.66x10^-10^ | Song (OCAC) ^145^ |
| rs7861573 | 9p22.2 | “ | 0.83 (0.78-0.88) | 3.61x10^-10^ | Song (OCAC) ^145^ |
| rs10756819 | 9p22.2 | “ | 0.83 (0.79-0.88) | 4.85x10^-12^ | Song (OCAC) ^145^ |
| rs1416742 | 9p22.2 | “ | 0.86 (0.82-0.90) | 1.74x10^-9^ | Song (OCAC) ^145^ |
| rs12379687 | 9p22.2 | “ | 0.82 (0.77-0.88) | 1.27x10^-8^ | Song (OCAC) ^145^ |
| rs4961501 | 9p22.2 | “ | 0.82 (0.77-0.87) | 8.49x10^-12^ | Song (OCAC) ^145^ |
| rs1339552 | 9p22.2 | “ | 0.85 (0.81-0.89) | 1.28x10^-10^ | Song (OCAC) ^145^ |
| rs10962692^†¶^ | 9 |  | 1.24 (1.20-1.29)  1.33 (1.27-1.38) | 9.2x10^-36^  3.4x10^-43^ | Song (OCAC) ^145^ |
| rs8170* | 19p13 | MERIT40,  BABAM1 | 1.18 (1.12-1.25) | 2.7x10^-9^ | Bolton (OCAC) ^146^ |
| rs2363956^†^ | 19p13 | ANKLE1 | 1.16 (1.11-1.21) | 3.8x10^-11^ | Bolton (OCAC) ^146^ |
| rs4808075^¶^ | 19 |  | 1.13 (1.10-1.16)  1.18 (1.14-1.22) * | 1.5x10^-17^  3.7x10^-24^ | Bolton (OCAC) ^146^ |
| rs2072590^†^ | 2q31 | HOXD3, HOXD1 | 1.20 (1.14-1.25) | 3.8x10^-14^ | Goode (OCAC) ^147^ |
| rs2665390^†^ | 3q25 | TiPARP | 1.24 (1.15-1.34) | 7.1x10^-8^ | Goode (OCAC) ^147^ |
| rs10088218^†^ | 8q24 | CMYC | 0.76 (0.70-0.81) | 8.0x10^-15^ | Goode (OCAC) ^147^ |
| rs9303542^†^ | 17q21 | SKAP1 | 1.14 (1.09-1.20) | 1.4x10^-7^ | Goode (OCAC) ^147^ |
| rs1400482^†¶^ | 8 |  | 1.18 (1.13-1.22)  1.27 (1.22-1.34) | 6.9x10^-16^  1.5x10^-24^ | Goode (OCAC) ^147^ |
| rs6755777^†¶^ | 2 |  | 1.11 (1.08-1.14)  1.13 (1.10-1.17) | 3.7x10^-14^  1.8x10^-14^ | Goode (OCAC) ^147^ |
| rs11782652 | 8q21 | CHMP4C | 1.19 (1.07-1.36) | 5.5x10^-9^ | Pharoah ^148^ |
| rs1243180 | 10p12 | MLLT10 | 1.10 (1.06-1.13) | 1.8x10^-8^ | Pharoah ^148^ |
| rs757210^†^ | 17q12 | HNF1B | 1.12 (1.08-1.17) | 9.6x10^-10^ | Pharoah ^148^ |
| rs7651446  r^2^ 0.61 with rs2665390 | 3q25 | TiPARP | 1.44 (1.35-1.53) | 1.5x10^-28^ | Pharoah ^148^ |
| rs1879586^¶^ | 17 |  | 1.13 (1.10-1.17) | 1.8x10^-13^ | Pharoah ^148^ |
| rs14462376^¶^ | 10 |  | 1.09 (1.06-1.12) | 7.8x10^10^ | Pharoah ^148^ |
| 8:82668818^¶^ | 8 |  | 1.16 (1.10-1.22)  1.19 1.13-1.26) | 1.7x10^-8^  2.4x10^-9^ | Pharoah ^148^ |
| Rs62274041^†¶^ | 3 |  | 1.45 (1.37-1.53)  1.59 (1.49-1.69) | 4.5x10^-38^  3.3x10^-46^ | Pharoah ^148^ |
| rs7705526^¶^ | 5p15.33 | TERT | 1.51 (1.36-1.67) | 1.3x10^-15^ | Bojesen ^149^ |
| rs10069690^†¶^ | 5p15.33 | TERT | 1.15 (1.11-1.20) | 1.25x10^-11^ | Bojesen ^149^ |
| rs17631303 | 17q21.31 | PLEKHM1, c17orf69, ARHGAP27, MAPT, WNT3, KANSL1 | HR 1.27 (1.17-1.38) | 1.4x10^-8^ | Couch (CIMBA) ^150^ |
| rs183211 | 17q21.31 | “ | HR 1.25 (1.14-1.38) | 3.5X10^-6^ | Couch (CIMBA) ^150^ |
| rs4691139^¶^ | 4q32.3 | TRIM61 | HR 1.20 (1.17-1.38) | 3.4x10^-8^ | Couch (CIMBA) ^150^ |
| rs56318008 | 1p36 | WNT4 | 1.11 (1.06-1.15)  1.12 (1.06-1.17)* | 8x10^-7^  6x10^-6^ | Kuchenbaecker (OCAC, CIMBA) ^151^ |
| rs58722170^¶^ | 1p34.3 | RSPO1 | 1.07 (1.03-1.11)  1.12 (1.07-1.17)* | 2x10^-4^  4x10^-7^ | Kuchenbaecker (OCAC, CIMBA) ^151^ |
| rs17329882^¶^ | 4q26 | SYNPO2 | 1.09 (1.06-1.13)  1.11 (1.07-1.16)* | 3x10^-7^  3x10^-7^ | Kuchenbaecker (OCAC, CIMBA) ^151^ |
| rs116133110 | 6p22.1 | GPX6 | 0.94 (0.91-0.97)  0.91 (0.87-0.94)* | 9x10^-5^  3x10^-7^ | Kuchenbaecker (OCAC, CIMBA) ^151^ |
| rs6355634 | 9q34.2 | ABO | 1.12 (1.08-1.16)  1.13 (1.08-1.18)* | 9x10^-9^  2x10^-7^ | Kuchenbaecker (OCAC, CIMBA) ^151^ |
| Chr17:29181 220:1  rs7405776^¶^ | 17q11.2 | ATAD5 | 0.90 (0.87-0.93)  0.90 (0.87-0.94)* | 1x10^-9^  2x10^-7^ | Kuchenbaecker (OCAC, CIMBA) ^151^ |
| 9:136138765  ^†¶^ | 9 |  | 1.11 (1.07-1.15)  1.13 (1.09-1.18) | 8.3x10^-10^  6.6x10^-11^ | Kuchenbaecker (OCAC, CIMBA) ^151^ |
| Rs6456822*^¶^ | 6 |  | 1.07 (1.04-1.11) | 3.1x10^-5^ | Kuchenbaecker (OCAC, CIMBA) ^151^ |
| 1:22470407^¶^ | 1 |  | 1.07 (1.03-1.11) | 4.3x10^-4^ | Kuchenbaecker (OCAC, CIMBA) ^151^ |
| rs752590^§¶^ | 2q13 | PAX8 | 1.34 (1.21-1.49) | 3.3 × 10^−8^ | Kelemen et al ^152^ |
| rs711830^§¶^ | 2q31.1 | HOXD3 | 1.30 (1.20-1.40) | 7.5 × 10^−12^ | Kelemen et al ^152^ |
| rs688187^§¶^ | 19q31.2 | IFNL3 | 0.67 (0.60-0.75) | 6.8 × 10^−13^ | Kelemen et al ^152^ |
| rs3744763^†^ | 17 | HNF1B | 1.13 (1.09-1.17) | 4.0 x 10^-10^ | Shen et al ^153^ |
| rs36092841^†^ | 17 | HNF1B | 1.13 (1.08-1.17) | 3.1x10^-9^ | Shen et al ^153^ |
| rs7405776^†¶^ | 17 | HNF1B | 1.13 (1.09-1.17) | 3.1x10^-10^ | Shen et al ^153^ |
| rs757210^†^ | 17 | HNF1B | 1.13 (1.09-1.17) | 3.2x10^-10^ | Shen et al ^153^ |
| rs4239217^†^ | 17 | HNF1B | 1.11 (1.07-1.16) | 2.6x10^-8^ | Shen et al ^153^ |
| rs11651755^^¶^ | 17 | HNF1B | 0.77 (0.70-0.84) | 1.6x10^-8^ | Shen et al ^153^ |
| rs61612821^†^ | 17 | HNF1B | 1.19 (1.13-1.26) | 1.1x10^-9^ | Shen et al ^153^ |
| rs11657964^†^ | 17 | HNF1B | 1.12 (1.08-1.16) | 5.3x10^-9^ | Shen et al ^153^ |
| rs7501939^†^ | 17 | HNF1B | 1.12 (1.08-1.16) | 4.8x10^-9^ | Shen et al ^153^ |
| rss11658063^†^ | 17 | HNF1B | 1.12 (1.08-1.17) | 1.8x10^-9^ | Shen et al ^153^ |
| rs1052587^†^ | 17q21.31 | MAPT | 1.12 (1.08-1.17) | 4.6x10^-8^ | Permuth-Wey ^154^ |
| rs12942666 | 17q21.31 | ARHGAP27 | 1.11 (1.07-1.15)  1.15 (1.11-1.20)* | 3.3x10^-8^  1.0x10^-9^ | Permuth-Wey ^154^ |
| rs2960000 | 17q21.31 | PLEKHM1 | 1.12 (1.08-1.16)  1.16 (1.12-1.20)* | 4.2x10^-9^  3.3x10^-10^ | Permuth-Wey ^154^ |
| rs2077606 | 17q21.31 | PLEKHM1 | 1.12 (1.08-1.16)  1.15 (1.12-1.19)* | 7.8x10^-9^  3.9x10^-10^ | Permuth-Wey ^154^ |
| rs7207826^¶^ | 17 |  | 1.11 (1.08-1.14)  1.14 (1.10-1.18) | 7.7x10^-13^  1.2x10^-14^ | Permuth-Wey ^154^ |
| rs17041869^¶^  (+br +pr ca) | 2q13 | BCL2L11 | 0.94 (0.93-0.96) | 5.1x10^-9^ | Kar et al ^155^ |
| rs7937840  (+br +pr ca) | 11q12 | INCENP | 1.05 (1.03-1.06) | 5.0x10^-9^ | Kar et al ^155^ |
| 11:61893972^¶^ | 11 |  |  |  | Kar et al ^155^ |
| rs1469713^¶^  (+br +pr ca) | 19p13 | GATAD2A | 0.96 (0.95-0.97) | 3.4x10^-10^ | Kar et al ^155^ |
| rs200182588  (+br ca) | 9q31 | SMC2 | 0.95 (0.94-0.97) | 8.9x10^-9^ | Kar et al ^155^ |
| rs8037137^¶^  (+br ca) | 15q26 | RCCD1 | 1.07 (1.05-1.10) | 9.1x10^-10^ | Kar et al ^155^ |
| rs4742903^¶^ | 9 |  | 1.06 (1.03-1.08)  1.07 (1.03-1.10) | 4.1x10^-5^  3.5x10^-5^ | Kar et al ^155^ |
| rs9870207^†¶^ | 3q28 | ? | 1.19 (1.12-1.27) | 4.5x10^-8^ | Phelan et al (OCAC, CIMBA) ^129^ |
| rs13113999^†¶^ | 4q32.3 | ? | 1.23 (1.14-1.32) | 4.7x10^-8^ | Phelan et al (OCAC, CIMBA) ^129^ |
| rs150293538^†¶^ | 8q21.11 | LINC01111 | 2.19 (1.65-2.90) | 2.0x10^-9^ | Phelan et al (OCAC, CIMBA) ^129^ |
| rs7902587^†¶^ | 10q24.33 | ? | 1.29 (1.18-1.41) | 4.0x10^-8^ | Phelan et al (OCAC, CIMBA) ^129^ |
| rs8098244^†¶^ | 18q11.2 | LAMA3 | 1.19 (1.12-1.27) | 3.9x10^-8^ | Phelan et al (OCAC, CIMBA) ^129^ |
| rs6005807^†¶^ | 22q12.1 | TTC28 | 1.17 (1.11-1.23) | 4.5x10^-9^ | Phelan et al (OCAC, CIMBA) ^129^ |
| rs112071820^§¶^ | 3q22.3 | ? | 1.29 (1.20-1.37) | 1.5x10^-13^ | Phelan et al (OCAC, CIMBA) ^129^ |
| rs555025179^‡¶^ | 5q12.3 | MAST4 | 1.18 (1.11-1.26) | 4.5x10^-8^ | Phelan et al (OCAC, CIMBA) ^129^ |
| rs320203^§¶^ | 9q31.1 | ? | 1.29 (1.18-1.41) | 1.7x10^-8^ | Phelan et al (OCAC, CIMBA) ^129^ |
| rs2165109^†¶^ | 2q13 | ACOXL | 1.09 (1.05-1.12) | 4.2x10^-8^ | Phelan et al (OCAC, CIMBA) ^129^ |
| rs9886651^†¶^ | 8q24.21 | PVT1 | 1.08 (1.05-1.11) | 3.5x10^-9^ | Phelan et al (OCAC, CIMBA) ^129^ |
| rs7953249^†¶^ | 12q24.31 | ? | 1.08 (1.06-1.06?) | 1.1x10^-9^ | Phelan et al (OCAC, CIMBA) ^129^ |

^†^ = serous ovarian cancer

^‡^ = endometrioid cancer

**^§^** = mucinous ovarian cancer

**^¶^ = Included in Phelan et al total 34 SNPs**
